# New insights into intestinal phages

**DOI:** 10.1038/s41385-019-0250-5

**Published:** 2020-01-06

**Authors:** R. Sausset, M. A. Petit, V. Gaboriau-Routhiau, M. De Paepe

**Affiliations:** 1 Micalis Institute, INRA, AgroParisTech, Université Paris-Saclay, 78350 Jouy-en-Josas, France; 2 Myriade, 68 boulevard de Port Royal, 75005 Paris, France; 3 Laboratory of Intestinal Immunity, INSERM UMR 1163, Institut Imagine, Paris, France; 4 Université Paris Descartes-Sorbonne Paris Cité, 75006 Paris, France

## Abstract

The intestinal microbiota plays important roles in human health. This last decade, the viral fraction of the intestinal microbiota, composed essentially of phages that infect bacteria, received increasing attention. Numerous novel phage families have been discovered in parallel with the development of viral metagenomics. However, since the discovery of intestinal phages by d’Hérelle in 1917, our understanding of the impact of phages on gut microbiota structure remains scarce. Changes in viral community composition have been observed in several diseases. However, whether these changes reflect a direct involvement of phages in diseases etiology or simply result from modifications in bacterial composition is currently unknown. Here we present an overview of the current knowledge in intestinal phages, their identity, lifestyles, and their possible effects on the gut microbiota. We also gather the main data on phage interactions with the immune system, with a particular emphasis on recent findings.

## Introduction

The human gut contains a large number of viruses, mostly bacteriophages, or phages, which infect bacteria. As other viruses, phages are classified according to their type of nucleic acid, capsid morphology - notably the presence or absence of a tail -  and the presence or not of an envelope. The genetic material of phages consists of double-stranded (ds) or single-stranded (ss) DNA or RNA, and their genome sizes range from ∼3.5 kb (e.g., ssRNA genome of *Escherichia coli* phage MS2) to ∼540 kb (dsDNA genome of *Prevotella* LAK phages). There is considerable diversity among phages, but 95% of them are non-enveloped tailed dsDNA phages, or *Caudovirales*. Within this group, the traditional differentiation into *Siphoviridae*, *Myoviridae*, and *Podoviridae* families, based on tail types, is not fully coherent with phylogeny, and therefore progressively abandoned. In addition, new phage types are constantly discovered, and classification is currently ongoing reorganization.

Phages are present in all microbial environments and the importance of phage predation on bacteria is evidenced by the large repertoire of bacterial anti-phage defence mechanisms. Anti-phage systems include cell-surface modifications that prevent phage recognition (phage multiplication is highly dependent on the proper selection of their target bacteria, which is achieved by the recognition of a specific structure on the bacterial surface^1^), but also abortive infection mechanisms that trigger cell death upon phage infection and restriction-modification or CRISPR–Cas systems that cleave invading phage genomes (reviewed in refs. ^2^ and ^3^).

The presence of phages in the intestine has been described only 2 years after their discovery by Twort,^4^ when d’Hérelle^5^ independently discovered phages, and their therapeutic potential, in the stools of patients with dysentery. Before the dawn of antibiotics, but also later on in the Soviet Union, phages have been utilized to treat a variety of intestinal infections, mainly cholera^6^ and dysentery.^7^ However, the success of these treatments has been variable and antibiotics proved to be both more efficient and cost-effective, leading to the almost abandonment of phage therapy in most countries (reviewed in ref. ^8^). With the rise of bacterial resistance to antibiotics, phage therapy has recently regained interest, fueling researches on applied but also basic phage biology. The relatively recent discovery of the influence of phages in aquatic bacterial ecosystems further explains the present bloom of phage studies.^9^ Finally, due to increased awareness of the importance of the gut microbiota in human health, a growing number of studies are addressing the roles of phages in the gut microbiota. Emerging views suggest that intestinal phages play important roles in health and disease by shaping the co-occurring bacteriome, but also by interacting directly with the human immune system.^10–12^ Several recent reviews have exhaustively reported different aspects of intestinal phage biology, such as its genetic diversity,^13,14^ bacterial resistance mechanisms, including CRISPR–Cas systems and other molecular mechanisms of phage–bacteria interactions,^2,3^ phage–bacterium antagonistic interactions in the gastro-intestinal tract (GIT),^15,16^ lysogeny,^11^ and phage interactions with the host immune system.^10,11,17^ Here we aim at giving a global view of current knowledge of phages in the GIT, emphasizing on new results, open questions, and technical difficulties of this rapidly growing field of research.

## Composition of the intestinal phageome

**Fig. 1:**
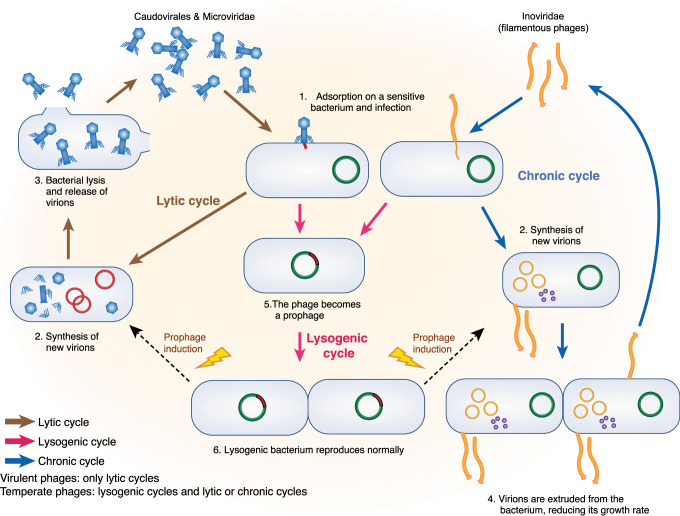
Phage life cycles. The production of new virions is realized either through lytic cycles for *Caudovirales* and *Microviridae* phages (left side of the figure, brown arrows) or through chronic infection in the case of filamentous phages, or *Inoviridae* (blue arrows). Both start with the recognition and infection of the targeted bacteria (1), followed by phage DNA replication and synthesis of new virions (2). In lytic cycles, new virions are released through bacterial lysis (3), while new virions of filamentous phages exit bacteria through a dedicated secretion apparatus, without bacterial lysis (4). Phages that reproduce only through lytic cycles are called virulent. By opposition,  some phages, called temperate phages, in addition to performing either lytic or chronic cycles, are able to perform lysogenic cycles (pink arrows), whereby they enter a dormant state in the infected bacteria, the prophage state (5). The prophage, either integrated within the bacterial genome or in an episomal state, is replicated with the bacterial chromosome as long as bacteria divide (6). In some bacteria, generally when submitted to a stress, the prophage is induced and the phage resumes a lytic or a chronic cycle.

Description of intestinal phages, either from a taxonomic or lifestyle point of view, is still in its infancy compared with that of intestinal bacteria, and encounters technical difficulties. First, viral genomes lack universal marker genes such as the 16SrRNA gene used for bacterial taxonomic assignment. Second, the genetic diversity of phages remains largely unknown, preventing sequence-based identification of most intestinal phages. Typically, 75% to 99% of sequences from intestinal phages do not produce significant alignments to any known viral genome.^13^ Finally, intestinal phages are very challenging to cultivate, notably because their bacterial hosts are mainly strict anaerobes that are difficult to grow. However, starting from 0.2 or 0.45 µm filtered fecal samples enriched in virions, shotgun deep sequencing has permitted access to the human free-phage content (which will be designated below virome or phageome, since it comprises mainly phages). Most phages appeared to be non-enveloped DNA viruses, either dsDNA *Caudovirales* or ssDNA *Microviridae*. In addition, a recent study indicates that ssDNA filamentous phages, or *Inoviridae*, that reproduce through chronic infection without killing their host (Fig. 1) might also constitute a significant fraction of the human gut virome.^18^ In contrast, RNA phages were found to be rare, if not completely absent, in the intestine.^19,20^

In the intestine as in other environments, phages can be distinguished on the basis of their lifestyle, independently of taxonomy (Fig. 1). Virulent phages essentially complete lytic cycles, whereby each infection is followed by virion production and host cell lysis. Yet, in some conditions such as nutritional stress, virulent phage multiplication can be halted for a long time. This phenomenon, called pseudolysogeny, is poorly described, but is suspected to exist in the intestinal environment (reviewed in ref. ^21^). Temperate phages, for their part, can use two very different lifestyles, the so-called “lysis–lysogeny choice”: infection is either followed by a lytic cycle, as with virulent phages, or by lysogeny, whereby the phage enters a dormant state and is called a prophage. In this state, the expression of most phage genes is repressed, preventing phage multiplication, but the phage genome is replicated passively along with the bacterial genome. The prophage can be either integrated in the bacterial chromosome or extrachromosomal, like a plasmid. Following specific cues described below, prophages can be activated, leading to phage lytic cycle and death of the previously lysogenic bacteria. In consequence, when considering intestinal phages, one should take into account both free-phages and prophages.

Using recent assembly procedures, tens to thousands of DNA phage contigs can be assembled per virome sample, depending on sequencing depth. Yet, the functional roles of phages in the gut ecosystem remain difficult to apprehend, notably because they belong to entirely new and still uncharacterized genera and even families, and their bacterial hosts are unknown. Several recent reviews have summarized the main characteristics of the new findings in this emerging field.^13,14,22^ Their conclusions about the phage community in the human gut are summarized below.

1. Most phages appear unique to each individual.^23–25^ The existence of a “core phageome”, i.e., a small number of phages shared between individuals, is under debate. A first study indicated that across 62 healthy individuals, 23 phage contigs (0.5% of all contigs) were shared by at least 50% of individuals and 132 contigs (3%) by at least 20% of individuals.^24^ More recently, another study found that no viral population (equivalent to viral species) was present in more than half of 132 samples from healthy individuals, and that only 1% was shared by over 20% of individuals.^24,25^ These differences depend on the criteria used to identify a given phage in a given virome sample and comparisons suffer notably from the lack of consensus on phage taxonomy. Recent remarkable progresses in genome-based phage taxonomy^26,27^ may enable to better define the most common phage “types” in the human microbiota in the near future.
2. Healthy individuals tend to conserve the same phages over time (tested over 1 year), especially the most abundant ones^23,28,29^ (Fig. 2). Persistent phages, i.e., phages that reside at least one year in a given individual, seem also to be more commonly shared than others. Thus, among persistent phages, 22 out of the 3639 viral clusters (0.6%; clusters might reflect genus level groupings) were shared by more than half of the 10 individuals tested.^29^

**Fig. 2:**
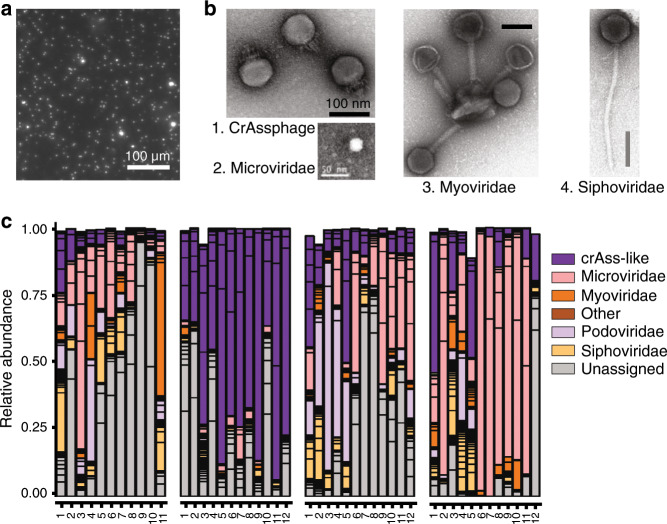
Main intestinal phage types. **a** Epifluorescence microscopy image of a human fecal filtrate. Virus-like particles (VLPs) appear as bright dots following Sybr-gold staining of DNA (M. De Paepe, unpublished results). **b** Transmission electronic microscopy images of major intestinal phage types. Scale bars are 100 nm except for the *Microviridae* virion, for which it is 50 nm. 1: CrAssphage PhiCrAss001 (*Podoviridae*), reproduced from ref. ^45^ 2: *Microviridae* virion isolated from surface water near a coastal aquaculture site, reproduced from ref. ^126^ 3 and 4: *Myoviridae* and *Siphoviridae* virions respectively, extracted from human intestinal contents. Several *Myoviridae* virions are bound to a membrane vesicle. Reproduced from ref. ^50^ Copyright *©* 2014 Elsevier Masson SAS. **c** Inter-individual virome specificity and conservation in four human subjects over 12 months. Each phage cluster, which roughly corresponds to a phage genus, is outlined in black. Reproduced from ref. ^29^
3. In the human gut, temperate phages constitute at least 20% to 50 % of free phages, depending on samples but also on the methodology, as temperate phages are difficult to distinguish from virulent ones. For example, a study focusing on the virome content of twins estimated the proportion of temperate phages on the basis of the proportion of phage contigs encoding an integrase gene, this gene being responsible for the integration of temperate phage genome into the bacterial chromosome.^23^ However, temperate phages do not necessarily encode an integrase gene, as many of them coexist with their host as autonomously replicating episomes, and such method gives only a lower bound of the proportion of temperate phages. The high abundance of temperate phages is compatible with the observation that among isolated gut bacteria, most strains are lysogens and often polylysogens, notably within the dominant *Bacteroidetes*, *Firmicutes*, *Actinobacteria*, and *Proteobacteria* phyla.^30–34^ Several metagenomic studies further suggest that the majority of intestinal bacteria carry prophages.^35,36^ Yet, estimations of prophage prevalence based on genomic analyses cannot distinguish “active” prophages from “defective” ones, i.e., those that have lost the capacity to resume a lytic cycle due to mutations. However, the presence of active prophages, which produce virions, has been demonstrated experimentally in almost all gut bacterial strain tested, suggesting that a significant proportion of prophages detected by genomic analyses are active.^33,34,37,38^ High prevalence of temperate phages in human microbiota could participate in the stability of the phageome over time, as populations of lysogenic bacteria constantly produce virions.
4. The identity of the hosts targeted by the phages is a crucial but largely unanswered question. In 2016, Edwards et al.^39^ compared different methods for host prediction and those that gave the best results were simple nucleotide similarity searches between phage and bacterial genomes, either with BLASTn or by searching the longest exact nucleotide match between a phage and a bacterial genome. With these methods, the correct hosts were predicted for 37% and 40%, respectively, of the 820 complete phages included in the test. Several studies have used CRISPR spacers to predict hosts,^29,39–41^ which led to very confident predictions but was restricted to hosts encoding CRISPR–Cas systems and in which phage infection was relatively recent. Between 4% and 13% of phages could be assigned to a host this way. Finally, the program WiSH bases its predictions on the similarity of the phage genome to that of its hosts. It uses a probabilistic approach that compares the composition in subsequences of nine nucleotides, or 9-mers, in phage and bacterial genomes, and reaches good prediction even for short 3 kb-long phage contigs, which is not the case for other methods. Using the same large data set as Paez-Espino et al.^41^, WiSH predicted a host at family level for 59% of the contigs.^42^ Using a mix of these approaches, recent studies indicate that the spectrum of the bacterial hosts of the dominant phages reflects the microbiome composition.^29,43^ Indeed, among 180 persistent phage clusters identified, about one third could be linked to a bacterial genus, all of them belonging to abundant taxa, such as *Faecalibacterium* and *Bacteroides*.^29^
5. In individuals consuming a western diet, two particularly prevalent and abundant phage taxa have been recently described, both infecting *Bacteroides* species (Fig. 2):
  *Gokushovirinae* are temperate phages belonging to the *Microviridae* family, with a small circular ssDNA genome, and a small virion of 30 nm of diameter.^31^ Given the nature of their genome, these virions can escape scrutiny or, on the contrary, be overestimated depending on the kit and treatments used prior to sequencing (discussed in ref. ^14^). Therefore, their exact contribution to the phageome remains to be more accurately estimated.
  CrAss-like phages constitute a completely new clade of related phages present in at least 50% of individuals from western cohorts. They can represent up to 90% of the phageome of a single individual. CrAss-like phages have a dsDNA genome of ~100 kb, a 90 nm diameter large head and a short tail (Fig. 2b). Their abundance has helped assembling many genomes of the clade, which presently span over four proposed subfamilies.^44^ One of them, CrAss001, infects *Bacteroides intestinalis*.^45^ Due to difficulties in cultivating the CrAssphages, their lifestyle is not clearly established yet, but the presence of an integrase in some of them suggests they may be temperate phages.^29^

In addition, a distinct prevalent phage taxon, called LAK phages, has recently been described in individuals from Tanzania and the region of Laksam, in Bangladesh. Their genomes were assembled directly from complete microbiota samples rather than using viral fractionation.^46^ Due to their remarkably large genome size (540 kb), they were coined “megaphages”, but their virions are not imaged yet. LAK phages have been shown to preferentially infect *Prevotella*, but their lifestyle is still unknown.

## Quantification of phages in the GIT

Defining the number of phages present in the GIT should help to predict their impact on bacteria. As already stressed, both free phages and prophages should be taken into account. Prophages are present in the majority of bacterial genomes and are thus approximately as numerous as bacteria in the GIT. Free phages, produced during lytic cycles, are generally enumerated as virus-like particles (VLPs). VLPs are nanoparticles that can be observed by epifluorescence microscopy following staining of nucleic acids (Fig. 2a). However, if such nanoparticles are mainly virions, some are membrane vesicles containing nucleic acid that are difficult to distinguish from virions by microscopy.^47^ In addition, immunologists often define VLPs as particles obtained by spontaneous assembly of viral structural proteins, antigenically indistinguishable from infectious viruses, but that do not contain the viral genome.^48^ Despite these restrictions, we will use indiscriminately virions and the microscopy-based definition of VLPs below. In most ecosystems, free phages outnumber bacteria by about tenfold.^49^ In contrast, the small number of available studies enumerating virions in human stools indicates that in healthy subjects, bacteria outnumber virions. Two studies reported VLP abundances ranging from 1 × 10^8^ to 2 × 10^9^ VLPs/g in stools,^50,51^ whereas bacterial concentration is thought to be 1 × 10^11^ bacteria/g.^52^ Recently, the concentration of virions was also estimated by spiking viral metagenomic samples with a known concentration of a specific virus, indicating that total viral loads may be between 2 × 10^8^ and 8 × 10^10^ viral genome copies per gram of stool.^29^ The ratio of free phages to bacteria that can be extrapolated is thus comprised between 1 and 0.001, overall suggesting lower phage-induced mortality in the human gut microbiota than in other microbial ecosystems. An alternative interpretation is that a large fraction of virions are “lost” in the GIT. Several factors may favor virion disappearance. First, virions bind irreversibly to their bacterial receptors with a very fast rate when receptor concentration is high.^53^ Such receptors can be present on the surface of susceptible bacteria, but also on the surface of resistant bacteria or even on other structures such as membrane vesicles or bacterial debris. In these last cases, binding of virions on their receptor results in their inactivation. As the bacterial concentration in the GIT is much higher than in most other microbial ecosystems, a higher part of virions could be lost by such a mechanism. For example, binding of virions on membrane vesicles is regularly reported in intestinal samples (Fig. 2b). Second, phages may be captured inside the mucus layer. Indeed, the Ig-like protein domain on the capsid of phage T4 was shown to increase by fourfold virion binding to mucins in vitro.^54^ As numerous intestinal phages possess similar motifs on their capsid proteins,^55^ it may account for the large number of virions detected in the intestinal mucus.^56^ Whether these mechanisms operate in the human microbiota remains to be investigated. Experiments in gnotoxenic mice (i.e. colonized with a limited number of bacterial strains), in which the majority of the susceptible bacteria were killed by phage, suggest that virion loss is highly dependent on individual phages considered. In some reports, free phage to bacteria ratios over 100 were observed in the feces of mice,^37,57^ whereas in others, ratios close to 1 were observed,^58,59^ suggesting either virion loss or very low viral amplification per infected bacteria, i.e., about one new virion produced per lytic cycle.

## Phage–bacterium interactions in the GIT

Phage–bacterium interactions in the gut microbiota are highly complex. First, phages often interact specifically with one single bacterial strain. Given bacterial diversity in the human gut, hundreds to thousands of phage–bacteria pairs may potentially interact at any time. Second, as discussed below, the intestinal environment seems to protect a fraction of the genetically susceptible bacterial populations from phage infection. Third, both antagonistic and mutualist interactions are possible. For example, bacteria can be killed by phages, either by infection followed by lytic cycle (predation) or upon prophage induction, but on the contrary, as we will see below, prophages and filamentous phages can provide benefits to their host. Finally, due to the high evolutionary capacities of phages and bacteria, phage–bacteria interactions may change very rapidly over time. Below we discuss the phage–bacterium interactions that have been demonstrated in the GIT and their impact on the gut microbiota composition.

### Prophage induction

Most prophages are highly stable, but environmental stressors or stochastic fluctuations in phage repressor concentrations can trigger their induction, i.e., resumption of the lytic cycle and subsequent lysis of the host bacteria. In general, cellular signals triggering prophage induction are DNA damages, via the destabilization of the repressor or master regulator of lysogeny. Quinolone antibiotics, which cause DNA double-strand breaks, are the most described prophage inducers in the GIT.^60^ In consequence, as *E. coli* stx prophages encode the shiga toxin, treatment of human shigatoxigenic *E. coli* infection with quinolones have significant adverse clinical consequences.^60^ Spontaneous induction rates, estimated in vitro to be between 10^−7^ and 10^−4^, were generally considered to be too low to negatively impact the lysogen’s fitness (reviewed in ref. ^61^). Yet, several pieces of evidence suggest that induction rates are globally higher in the murine GIT than in classical in vitro growth cultures, due to more frequent activation of the DNA damage response (SOS response).^58,62,63^ In the case of *Lactobacillus reuteri*, SOS activation was proposed to result from the activation of specific bacterial metabolic pathways in the GIT.^62^ Conflicting results were reported concerning the possible increase or, on the contrary, decrease of *E. coli* stx prophage induction rates by gut metabolites such as nitric oxide or bile salts (reviewed in ref. ^64^). Bile salts were also shown to induce some *Salmonella* prophages^.^^65^. Besides, intestinal inflammation increases the induction of a *Salmonella* prophage in mice.^66^ Finally, one has to mention the recent discovery of quorum-sensing pathways in phages of *Enterococcus faecalis* and *Vibrio cholerae*, suggesting that some prophages could regulate their induction rate in response to bacterial concentration.^67,68^ Altogether, these results suggest that in the GIT, prophage induction might constitute a significant burden for their bacterial host, and alter microbiota composition. Indeed, it has been shown in mouse models that a high prophage induction rate modifies the equilibrium between bacterial strains by disfavoring lysogens.^38,58^

### Lysogenic conversion

Some prophages compensate their detrimental effects on their host by providing them beneficial traits that augment their fitness and can confer completely novel phenotypes, a phenomenon known as “lysogenic conversion.” Examples of lysogenic conversion are numerous and recently reviewed by Taylor et al.^69^ and Wahl et al.^70^ in *Salmonella*. These new phenotypes comprise immunity to phage super-infection, resistance to other phages, tolerance to various stresses, pathogenicity, and, very rarely, antibiotic resistance. With respect to antibiotic resistance genes (ARGs), their presence within phage genomes is debated. Most reports are flawed by either excessive bacterial DNA contamination of the virome samples^71–74^ or inappropriate thresholds used for the similarity search against ARG databases.^75^ When both pitfalls were eliminated, ARG were rare in phage genomes (around or <1 gene among 10^5^ genes analyzed, on phage genomes and phage metagenomic contigs respectively),^76^ confirming an ancient observation.^77^ Further illustrating the potential of prophages to confer fitness advantages to their bacterial hosts, a very recent study analyzing sponge-associated virome suggested that some prophages encoding ankyrin-repeat proteins may provide anti-inflammatory properties to their bacterial host and foster host–microbe symbiosis.^78^

Independently of lysogenic conversion, a few prophages can function as genetic switches, a phenomenon known as “active lysogeny” (reviewed in ref. ^79^). In active lysogeny, prophage excision does not result in lytic replication, but restores the integrity of the bacterial gene or operon in which the prophage was integrated. For the intracellular pathogen *Listeria monocytogenes*, active lysogeny was shown to facilitate bacterial escape from the phagosomes.^80^ In a third situation, the production of virions itself benefits the lysogen population (reviewed in ref. ^61^). For example, production of filamentous phage particles by *Neisseria meningitidis* promotes bacterial aggregation in vitro via the formation of bundles of phage filaments, supposedly increasing the bacterial colonization of the nasopharynx.^81^

Due to the beneficial traits conferred to bacteria by prophages, temperate phages are often considered as mutualists rather than parasites of their bacterial host, and the prevalence of lysogeny is often interpreted as an evidence that prophages increase the selective fitness of their hosts (reviewed in ref. ^82^). However, if benefits conferred by prophages to pathogenic bacteria are well demonstrated, to the best of our knowledge, there is no direct demonstration that lysogenic conversion increases the colonization ability of a gut commensal in vivo, except very transiently by phage killing of a closely related strain.^58,83^ Alternatively, the prevalence of lysogeny may reflect regular lysogenization of bacteria by temperate phages, as evidenced in the GIT of animals,^58,66,84,85^ and even in the human gut microbiota.^86^

### Predation

**Fig. 3:**
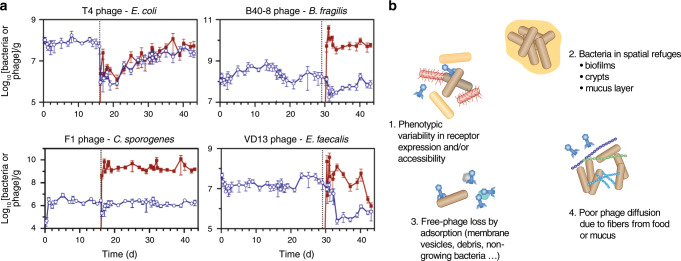
Phage-bacterium interactions in the GIT. **a** Illustration of the variability in the populations of phages and targeted bacteria in feces of gnotobiotic mice, reproduced from ref. ^57^ Germ-free mice were colonized with ten bacterial strains representing the major phyla of the human gut microbiota (*Clostridium sporogenes, E. faecalis, Bacteroides fragilis, B. ovatus, B. vulgatus, Parabacteroides distasonis, Klebsiella oxytoca, Proteus mirabilis, E. coli Nissle 1917*, and *Akkermansia muciniphila*). Dotted lines indicate the time of addition of 2 × 10^6^ PFU of a virulent phage targeting one bacterial strain exclusively. Bacteria and phages were quantified by quantitative PCR (blue and red lines, respectively). In the case of *E. coli*, after a rapid drop in bacterial population after T4 phage administration, phage and bacteria coexist without selection of phage-resistant mutants. In the cases of *C. sporogenes* and *B. fragilis*, bacterial populations are transiently affected by phage administration, and phages reach very high concentrations. Finally, in the case of *E. faecalis*, the bacterial population drops and does not recover its initial level over time, due to a defect of colonization by phage-resistant mutants. **b** Hypotheses for the co-existence of phage and susceptible bacteria in the GIT. (1) Important bacterial phenotypic variability, e.g., in phage receptor expression or growth rate, would render some bacteria phage-susceptible, whereas other genetically identical bacteria would be resistant. (2) Spatial heterogeneity of the environment (such as intestinal crypts, biofilm-like structures on food debris, or the inner part of the mucus layer) would create a refuge for bacteria and prevent access to phages. (3) Important loss of virions due to specific and nonspecific adsorption would sufficiently lower free-phage concentration to protect bacteria. (4) The presence of numerous long chain carbohydrates fibers from food and mucus would hinder phage diffusion, preventing them to adsorb on bacteria. None of these mechanisms has been formally demonstrated in the GIT.

A large body of information on phage predation in the GIT comes from animals with a modified microbiota, either gnotobiotic or treated by antibiotics, and colonized with specific bacterial strains and their associated phages. In all cases, phages multiplied successfully in the GIT. Yet, the decrease in the targeted bacteria is very variable (Fig. 3a).^57,59,87,88^ In some experiments, phage-resistant bacterial mutants were observed. However, and in contrast with results obtained in vitro, these mutants never entirely replaced the susceptible bacteria.^37,57,89^ In other cases, phage-mediated bacterial mortality was relatively limited and neither depletion of the bacterial population, nor selection of phage-resistant mutants were observed.^57,59,87,88^ Overall, these data suggest that the intestinal environment provides bacteria with spatial refuges or alternative resistance mechanisms, such as phenotypic resistance (Fig. 3b). For example, bile salts were shown to inhibit the infection of *E. coli* by several phages in vitro, possibly via the repression of a phase-variable cell-surface protein.^90^ Variability in phage resistance can also result from spatial heterogeneity in the bacterial population driven by gradients of abiotic factors, such as pH and oxygen, but also of molecules such as mucins, bile acids, and short-chain fatty acids. These gradients can modify the physiology of bacteria and, consequently, their susceptibility to phages, as shown ex vivo in Maura et al.^91^ Fluxes may also prevent phages to access some compartments, such as intestinal crypts.^15,91^ Finally, the crowded environment of the GIT may hamper phage diffusion (reviewed in refs. ^92^ and ^93^). Overall, most current reports point to a transient effect of phages on the population sizes of targeted bacteria in the GIT microbiota of animal models. Nevertheless, successful phage therapy trials realized before the 70s against intestinal bacteria indicate that phages may efficiently reduce bacterial loads in the human GIT.^5–7^

Another related question is the importance of phage-mediated mortality in a “natural” microbiota, i.e., without the artificial ingestion of high phage doses. At the timescale of evolution, phage killing left traces in the genomes of intestinal bacteria in the form of a large repertoire of anti-phage systems and highly diverse CRISPR spacer sequences.^40^ However, such traces of phage-mediated selection could result from a low level of phage-induced mortality, as even a small selective advantage can lead to allele fixation if sufficient time is given. At a shorter ecological timescale, there are also several evidences of significant natural phage-mediated bacterial mortality in humans and animals. First, a study by Seed et al.^94^ strongly suggests that mutations conferring phage resistance to *V. cholerae* occurred and were selected in two patients during cholera infection. In addition, metagenomic studies conducted on conventional animal’s feces showed a correlation between increases in specific phages and reductions in particular bacterial taxa, but without definitive proofs of direct interactions.^35,95^

Lastly, an interesting but poorly investigated phage–bacteria antagonistic interaction in the GIT is the potential ability of some phages to infect simultaneously several bacterial strains belonging, or not, to the same species. Although a large amount of work has indicated that most phages infect only a few strains of the same bacterial species, several clues suggest that intestinal phages might be more promiscuous than usually assumed. First, a phage infecting *Faecalibacterium prausnitzii* was shown to also infect *Blautia hansenii*, a species only distantly related to *F. prausnitzii*.^33^ Second, metagenomic analyses of CRISPR spacers have suggested that some phages may have a broad spectrum of hosts in the human gut.^29^ In addition, phage evolution resulting in the ability to infect a new host (host jump) was reported in a mouse model of coevolution in the GIT.^89^

In conclusion, phage–bacteria interactions in the GIT appear to be highly diverse, involving partial bacterial resistance, fast evolution of phages and bacteria, lysogenization and probably indirect cascading effects on several bacterial species and their phages.

## Impact of phages on host through gut microbiota modulation

### Phage therapy of intestinal disease infections

The complex relationship between phages and bacteria probably explains the relative unpredictability of phage therapy trials against intestinal pathogens. Phage therapy was nearly abandoned in most western countries 60 years ago given its unpredictable outcomes and the efficacy of newly discovered antibiotics. The compassionate use of phage therapy has been extensively practiced, though, in several countries, notably in Poland. The Eliava Institute, in Georgia, has been routinely treating various infections, and notably gut infections, with phage cocktails for more than a century, but no evaluation is available. An evaluation of Polish treatments reported symptomatic amelioration and clinical recovery in 40 and 18% of patients, respectively, which is quite encouraging for patients who previously failed to respond to antibiotic treatments.^96^ The mounting incidence of severe infections by multi-drug resistant bacteria, along with recent progresses in phage biology has prompted re-evaluation of phage therapy. A randomized controlled clinical trial was recently conducted against *E. coli* diarrhea in Bangladesh. It was unfortunately unsuccessful. Yet, it was subsequently shown that *E. coli* was probably not the cause of the diarrhea.^97,98^ This large scale study conducted up to stage III nevertheless permitted to establish the innocuous character of phage cocktails for humans.^98^ Besides classical phage therapy with defined phage mixtures, transfer of fecal filtrates containing phages but no bacteria, was proposed as an alternative to fecal microbiota transplantation (FMT) for *Clostridium difficile* infection.^99^ The transfer of fecal filtrates to five patients with chronic *C. difficile* infection was successful in all patients, suggesting that phages present in the fecal filtrates mediate many of the effects of FMT, but the mechanisms remain unknown.^99^

### Impact of phages on individuals with a healthy microbiota

Beyond phage therapy, given the numerous interactions between intestinal bacteria and the host, it is important to evaluate the extent of phage-induced modifications of microbiota functionality. First, as discussed previously, prophages modify numerous bacterial phenotypes, and notably virulence, thereby impacting host–bacteria interactions^69,70,78^. In particular, the ankyphages recently discovered in sponges might contribute to promote bacterial protection from the eukaryotic immune system.^100^ Indeed, transformed *E. coli* bacteria expressing a phage ankyrin protein better resisted murine bone-marrow-derived macrophage phagocytosis and induced a diminished inflammatory cytokine response in these macrophages. Interestingly, phage contigs encoding similar ankyrin proteins were found in various host-associated environments, including human oral and gut viromes, suggesting some wide-range phenomenon.^100^

Second, beyond lysogeny, phages can impact the mammalian host physiology through microbiota modulation, by depleting bacterial species important for homeostasis. However, as discussed previously, in animal models, phages never fully eradicate bacterial species in the gut, due to partial protection of bacteria and fast evolution of resistant bacterial mutants. Effects of phages on microbiota composition at the species level are therefore expected to be transient, unless they act in conjunction with other factors, such as other members of the microbiota. Studies aiming at detecting cascading effects on bacteria not targeted by the phage introduced have brought contrasting results. Studies in gnotobiotic rats and mice showed that beyond phage-mediated targeted knockdown of susceptible species in the gut, the concentration of non-targeted bacteria could be impacted, notably through modification of bacterial interactions.^57,101^ However, other studies in conventional animals showed no modification of the non-targeted microbiota.^102,103^ Similarly, a modification of the human microbiota composition has been observed upon oral administration of phages in one study,^104^ but not in another involving children.^105^ Such differences could be related to the difficulty of attributing shifts in microbiota composition to phages or to its natural temporal variability.

Phages could also impact indirectly the host if phage resistance affects the ability of bacteria to colonize the gut and to interact with their host. Indeed, many bacterial structures used as phage receptors, such as lipopolysaccharides, porins, flagella, fimbriae and pili, play a key role in bacterial colonization, growth, persistence, virulence and recognition by the host's immune system in the GIT (reviewed in refs. ^2,16^). For example, mutations in porins such as TonB or LamB, conferring phage resistance in *E. coli*, can diminish the nutritional competence of the bacteria, possibly affecting its gut colonizing ability. In particular, phage resistance was shown to negatively impact the ability of bacteria to develop antibiotic resistance, as bacterial mutants resistant to phage were more sensitive to antibiotics. For example, mutations in *Pseudomonas* conferring in vitro phage resistance affected a porin involved in multi-drug efflux,^106^ whereas in the case of *E. faecalis*, *epa* mutations changing its exopolysaccharide composition rendered the strain more sensitive to cell-wall-targeting antibiotics in mice.^107^ Moreover, several *E. faecalis epa* mutations could simultaneously confer phage resistance^107,108^ and defective intestinal colonization in mice.^109^ Indeed, a promising type of phage therapy consists in selecting phages on their faculty to favor the emergence of phage-resistant bacterial mutants that become antibiotic sensitive.^110^ Phages may also indirectly benefit their mammalian host by increasing the diversity of microbial communities,^16,111,112^ a known factor of stability of the gut microbiota.^113^ Finally, some phages can perform generalized transduction, permitting the transfer of bacterial DNA from an infected cell to another. Some data indicate that a substantial fraction of both temperate and virulent phages can perform such transduction (reviewed in refs. ^114^), so that this phenomenon may take place regularly in the GIT microbiota, participating in bacterial evolution.

### A role of phages on dysbioses?

Several studies have investigated correlations between virome composition and the physio-pathological state of individuals. One study notably reported a global shift in virome composition in patients with inflammatory bowel disease (IBD), either Crohn’s disease or ulcerative colitis, compared with household controls.^115^ Whether such shift implies that phages are actively involved in disease, or simply reflects the shift in microbiota composition remains to be shown. Nonetheless, re-analysis of the data focusing on the temperate phages of *F. prausnitzii*, a species generally depleted in IBD patients, showed that two *F. prausnitzii* phages were significantly more prevalent in IBD samples and two others were significantly more abundant,^33^ suggesting enhanced temperate phage-mediated mortality of *F. prausnitzii* in IBD. One may speculate that the inflammatory environment of the gut might increase prophage induction, as shown in *Salmonella*,^66^ thereby promoting bacterial lysis and aggravating dysbiosis, and reinforcing the inflammatory loop.

## Interactions between intestinal phages and the mammalian immune system

**Fig. 4:**
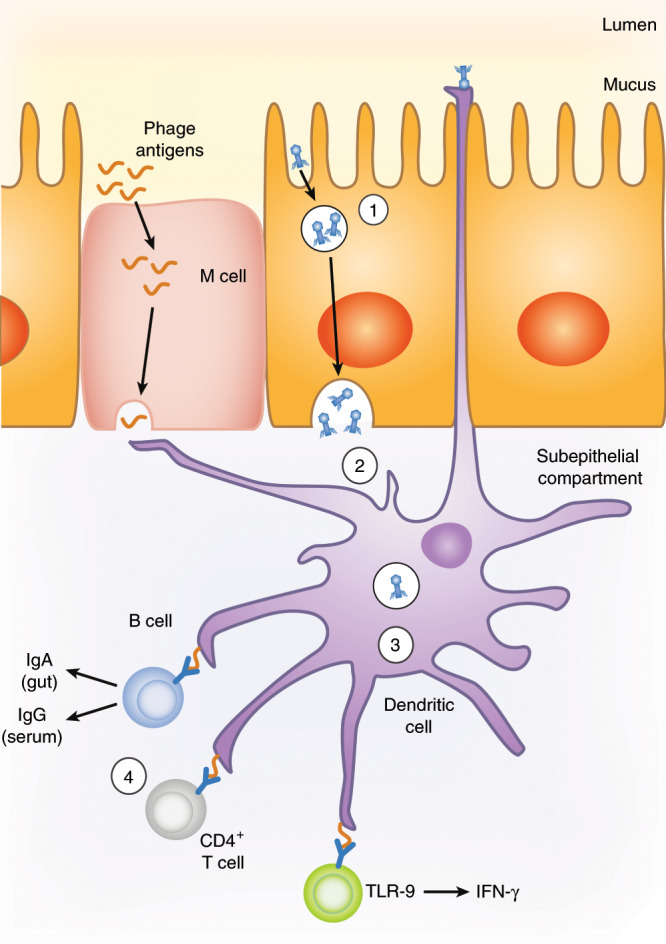
Potential interactions between phages, epithelial cells, and host immune cells in the intestine. Phage tropism for the mucus may promote penetration of phages within the body through endocytosis and transcytosis in intestinal epithelial cells (1), or through sampling by dendritic cells (2). Dendritic cells endocytose phages, may be captured in the intestinal lumen via extended dendrites, or exocytosed in the subepithelial compartment. Once endocytosed, phage nucleic acids can trigger TLR pathways, notably TLR9-dependent pathways (3), and stimulate adaptive immune responses (4). Although mechanisms of B and T-cell activation by phages are not fully elucidated, recent studies showed that activation of B cells leads to the secretion of phage-specific antibodies, both in the intestine and in the systemic compartment. In addition, activation of T cells in the Peyer’s patches and mesenteric lymph nodes results in production of cytokines, such as IFN-γ.

In addition to their indirect impact on hosts immune responses through changes in microbiota, phages may also interact directly with the host immune system and trigger immune responses (Fig. 4). Whether intestinal phages disseminate outside the gut lumen and interact with immune cells in the intestinal mucosa or at distant sites is a first matter of debate. Some studies in rodents and humans stated that no or few phages were detected in the bloodstream after oral administration. In contrast, others demonstrated effective dissemination, largely correlating with the dose of ingested phages^116^ (and reviewed in refs. ^11,12^). In addition, phage-neutralizing antibodies against naturally occurring intestinal phages have been detected in the sera of different mammalian species, indicating phage contact with host immune cells (reviewed in ref. ^17^). A recent study further showed that repeated oral exposure to high doses of the *E. coli* phage T4 induced simultaneously a specific protective secretory IgA response in the gut and a serum IgG response.^116^ Overall, these data suggest that phages can translocate through the mouse gut epithelium and activate the host immune system both locally and systemically. Translocation may occur across epithelial cells. Accordingly, recent in vitro data showed oriented phage transcytosis across several epithelial cell layers.^117^ Binding of phages to mucin glycoproteins^54^ may further promote contact with epithelial cells and transcytosis. Alternatively, phage uptake may involve dendritic cells. Indeed, dendritic cells exposed in vitro to phage particles were shown to efficiently phagocytose these particles^118–120^ and dendritic cells are thought to extend dendrites through the intestinal epithelium and thereby sample luminal bacteria and particles.^121^ However, the incidence of this phenomenon seems rare in the steady state, suggesting that luminal phage uptake by dendritic cells may be a relatively rare event.

Although more work is required to establish how phages can get access to host immune cells in vivo, recent studies have analyzed their interactions with host intracellular immune pathways and their capacity to trigger immune responses in vitro and in vivo. Following the unexpected observation that the treatment of mice with a cocktail of antiviral drugs aggravated dextran sulfate sodium (DSS)-induced colitis, Yang et al.^122^ suggested that gut resident viruses, a large fraction of which being phages, protect against intestinal inflammation via a mechanism involving TLR3 and TLR7-dependent production of interferon (IFN)-β. In this study, it is however difficult to assign a role to phages in TLR triggering as the antiviral drugs used target only eukaryotic viruses. Moreover, antiviral treatment was associated with changes in the microbiota, which may have also contributed to stimulate protection. Three other studies suggest that phages also directly interact with innate immune cells. Van Belleghem et al.^123^ observed that in vitro incubation of peripheral blood monocytes with purified *Staphylococcus aureus* or *Pseudomonas aeruginosa* phages induced a transcriptional response in monocytes, and notably enhanced the transcription of interleukin (IL)-1, IL-6, and tumor necrosis factor (TNF). Strengthening this in vitro study, Gogokhia et al.^12^ observed the expansion of IFN-γ-producing CD4^+^ T cells and of CD8^+^ T cells in the Peyer’s patches of germ-free mice orally treated with a lipopolysaccharide-free fraction of purified *E. coli* phages. They further showed that dendritic cells incubated with phages or phage-derived DNA could produce several cytokines including IL-12, IL-6, and IL-10, and stimulate CD4^+^ T-cell production of IFN-γ through a TLR9-dependent, but TLR3-independent, signal. In contrast with the effect of eukaryotic viruses reported by Yang et al.^122^ oral administration of the phage cocktail aggravated DSS colitis in a TLR9- and IFN-γ-dependent manner.^12^ Noticeably, the authors made a parallel with their observations in patients with ulcerative colitis treated by fecal transplantation, showing that the relative intestinal abundance of *Caudovirales* bacteriophages was higher in patients who failed to respond than in those who responded to this treatment.^12^ A second recent study provided evidence of TLR3-triggering by a filamentous phage present in the *P. aeruginosa* strains infecting chronic human wounds.^124^ Activation of TLR3 required endocytosis of phages by immune cells and, much surprisingly, neo-synthesis of phage RNA into the immune cell, which resulted in the production of type I IFN. This cytokine, in turn, inhibited the production of TNF by macrophages, thereby impairing phagocytosis and bacterial clearance, and delaying wound healing.^124^ Interestingly, this study suggests that phage uptake by mammalian immune cells, rather than simple cell-surface interactions, may be necessary to trigger host immune responses.

Overall, a limited set of data suggests that phages may exert a direct effect on immune cells. More work is however needed to define whether the effects observed in vitro or in vivo upon oral gavage with large amounts of purified virions can be recapitulated in more physiological conditions in the gut and how these effects may interfere or synergize with the changes induced by phages in the composition of the microbiota to impact mammalian health and disease, both in and out of the intestine.

## Conclusions

During the last decade, viral metagenomics has shed light on the taxonomic composition and dynamics of the viral component of the gut microbiota. Deep sequencing and novel assembly methods have allowed the description of completely new phages. These approaches have notably revealed that the virome composition is highly variable, with only a small fraction of phages shared among individuals. Not surprisingly, the most abundant viruses were found to infect *Bacteroides* and *Clostridiales* species that are dominant in the microbiota. In parallel, experiments in animals with a simplified microbiota allowed to explore phage–bacteria antagonistic interactions in the gut and have uncovered variable outcomes. For some phage–bacteria pairs, the intestinal environment somehow protects bacteria and only a small fraction of genetically susceptible bacteria are killed by their specific phages. In other cases, most of the phage-susceptible bacterial population is replaced by resistant mutants within a few days, indicating very efficient phage infection. Therefore, which outcome will prevail in complex natural microbiota remains difficult to predict. Traces of phage predation can be found in gut bacterial genomes, but these traces could result from a low level of phage-induced mortality. A low level of phage predation could nevertheless be crucial to shape microbiota composition and functionality, by affecting bacterial evolution through horizontal gene transfer, but also by promoting bacterial diversity. At shorter ecological timescales, the impact of phages is less well established. In particular, the role of phages in the dysbioses that accompany various pathological conditions remains poorly defined. Thanks to recent progresses in the determination of phage–bacteria pairs, longitudinal studies can now be undertaken to identify possible relationships between temporal shifts in bacteria and their associated phages and to delineate whether phages may contribute to dysbiosis and disease or, on the contrary, help to maintain microbiota stability by preserving bacterial diversity.

Further studies are also needed to substantiate possible direct interactions of phages with immune cells and to define whether and how such direct effects may modify the composition of the bacterial microbiota and influence host health or disease. Defining the mechanisms that determine the outcome of phage–bacteria interactions in the gut is particularly instrumental in the perspective of phage therapy, which, to cite Brüssow,^125^ “is without doubt an interesting approach to the antibiotic resistance problem and merits intensified research to get out of the fruitless confrontation between enthusiasm from the East and lingering Western skepticism”.
