# Correction: New insights into intestinal phages

**DOI:** 10.1038/s41385-020-0260-3

**Published:** 2020-01-31

**Authors:** R. Sausset, M. A. Petit, V. Gaboriau-Routhiau, M. De Paepe

**Affiliations:** 1 Micalis Institute, INRA, AgroParisTech, Université Paris-Saclay, 78350 Jouy-en-Josas, France; 2 Myriade, 68 boulevard de Port Royal, 75005 Paris, France; 3 Laboratory of Intestinal Immunity, INSERM UMR 1163, Institut Imagine, Paris, France; 4 Université Paris Descartes-Sorbonne Paris Cité, 75006 Paris, France

**Correction to**: *Mucosal Immunology* 10.1038/s41385-019-0250-5; published online 6 January 2020.

The original version of this Article contained an error in the author affiliations.

Affiliation [2] incorrectly read ‘Micalis Institute, INRA, AgroParisTech, Université Paris-Saclay, 78350 Jouy-en-Josas, Myriade, Paris, France’. Instead, it should read ‘Myriade, 68 boulevard de Port Royal, 75005 Paris, France’.

This has now been corrected in both the PDF and HTML versions of the Article.

